# Biguanides sensitize leukemia cells to ABT-737-induced apoptosis by inhibiting mitochondrial electron transport

**DOI:** 10.18632/oncotarget.9843

**Published:** 2016-06-06

**Authors:** Juliana Velez, Rongqing Pan, Jason T.C. Lee, Leonardo Enciso, Marta Suarez, Jorge Eduardo Duque, Daniel Jaramillo, Catalina Lopez, Ludis Morales, William Bornmann, Marina Konopleva, Gerald Krystal, Michael Andreeff, Ismael Samudio

**Affiliations:** ^1^ Grupo de Terapia Celular y Molecular, Pontificia Universidad Javeriana, Bogotá, Colombia; ^2^ Programa de Investigación e Innovación en Leucemia Aguda y Crónica (PILAC), Bogotá, Colombia; ^3^ Centro Oncológico de Antioquia, Medellín, Colombia; ^4^ Grupo de Terapia Regenerativa, Universidad de Caldas, Manizales, Colombia; ^5^ Department of Experimental Therapeutics, The University of Texas MD Anderson Cancer Center, Houston, TX, USA; ^6^ Section of Molecular Hematology and Therapy, The University of Texas MD Anderson Cancer Center, Houston, TX, USA; ^7^ Terry Fox Laboratory, British Columbia Cancer Agency, Vancouver, Canada; ^8^ Centre for Drug Research and Development, Vancouver, Canada

**Keywords:** metformin, phenformin, leukemia, mitochondria, ABT 737

## Abstract

Metformin displays antileukemic effects partly due to activation of AMPK and subsequent inhibition of mTOR signaling. Nevertheless, Metformin also inhibits mitochondrial electron transport at complex I in an AMPK-independent manner, Here we report that Metformin and rotenone inhibit mitochondrial electron transport and increase triglyceride levels in leukemia cell lines, suggesting impairment of fatty acid oxidation (FAO). We also report that, like other FAO inhibitors, both agents and the related biguanide, Phenformin, increase sensitivity to apoptosis induction by the bcl-2 inhibitor ABT-737 supporting the notion that electron transport antagonizes activation of the intrinsic apoptosis pathway in leukemia cells. Both biguanides and rotenone induce superoxide generation in leukemia cells, indicating that oxidative damage may sensitize toABT-737 induced apoptosis. In addition, we demonstrate that Metformin sensitizes leukemia cells to the oligomerization of Bak, suggesting that the observed synergy with ABT-737 is mediated, at least in part, by enhanced outer mitochondrial membrane permeabilization. Notably, Phenformin was at least 10-fold more potent than Metformin in abrogating electron transport and increasing sensitivity to ABT-737, suggesting that this agent may be better suited for targeting hematological malignancies. Taken together, our results suggest that inhibition of mitochondrial metabolism by Metformin or Phenformin is associated with increased leukemia cell susceptibility to induction of intrinsic apoptosis, and provide a rationale for clinical studies exploring the efficacy of combining biguanides with the orally bioavailable derivative of ABT-737, Venetoclax.

## INTRODUCTION

Acute leukemia remains largely incurable due to reappearance of chemoresistant blasts, even though most patients achieve a complete remission after first line induction and consolidation chemotherapy [reviewed in 1, 2]. It appears likely that the leukemic bone marrow provides a privileged sanctuary that shields quiescent leukemic progenitors from chemotherapy-induced cell death, with mesenchymal stromal cells (MSC) playing a critical role in chemoprotection via inducing complex genetic and signal transduction alterations in leukemia cells [reviewed in 3]. While other stromal cell types have been implicated, such as adipocytes [4] and osteoblasts [5], much remains to be understood regarding the precise cellular and molecular mechanisms that orchestrate the chemoresistant phenotype of leukemic cells within the bone marrow.

Mitochondrial uncoupling – a short circuit in the electrochemical gradient of the mitochondrial membrane – promotes resistance to intrinsic apoptosis in leukemia cells, in part via antagonism of bax/bak oligomerization [6, 7]. Moreover, this metabolic phenotype, originally reported in leukemia cells cultured on MSC feeder layers [6], results in decreased entry of pyruvate into the Krebs cycle and a shift to fatty acid oxidation (FAO) to support oxygen consumption, presumably permitting utilization of glucose carbon skeletons for the generation of biomass [8]. Intriguingly, pharmacologic inhibition of FAO using etomoxir – a substituted 2-oxirane-carboxylic acid that inhibits carnitine palmitoyl CoA transferase 1 – rapidly and almost completely inhibited oxygen consumption and sensitized leukemia cells to induction of apoptosis by the bcl-2 inhibitor ABT-737, in vitro and in xenograft models [7], substantiating the idea that mitochondrial oxidative metabolism supports leukemia cell survival. Since the clinical derivative of ABT-737 (ABT-199, Venetoclax; Abbvie) has been recently approved for the treatment of relapsed/refractory CLL with 17p deletion [9, 10], it is tempting to speculate that pharmacological inhibition of FAO using etomoxir could further improve the efficacy of bcl-2 inhibition in leukemia patients. However, the long term cytotoxic effects of etomoxir, its elevated cost, and its lack of approval for clinical use in most countries hinder its development as a therapeutic component for the treatment of leukemia.

The antidiabetic agent Metformin has chemopreventive and direct antitumor properties [reviewed in 11], and several ongoing clinical studies around the world are using this agent alone or in combination with chemotherapeutic schemes to determine prospectively its safety and efficacy in the treatment of human cancer [reviewed in 12]. Mechanistically, Metformin activates the AMP-dependent kinase (AMPK), either via the tumor suppressor kinase LKB1 [13], or by promoting an increase in AMP:ATP ratios [14]. Activated AMPK can in turn phosphorylate and activate TSC2, a negative regulator of the mammalian target of rapamycin (mTOR) [15]. While it is currently unclear how Metformin activates LKB1, it has been shown that Metformin can increase the AMP:ATP ratio as a result of moderate inhibition of the electron transport chain at the entry point of NADH, viz-a-viz mitochondrial complex I [14, 16]. Interestingly, while activation of AMPK and subsequent inhibition of mTOR-induced signaling has been suggested to mediate the antitumor effects of Metformin [reviewed in 17], AMPK-independent growth inhibitory properties of this agent in tumor cells have also been described [18–21], suggesting that antagonizing electron transport *per se* may be cytostatic or cytotoxic to cancer cells.

Here we report that pharmacologic inhibition of electron transport with Metformin, the related biguanide Phenformin, or rotenone sensitizes leukemia cells to induction of intrinsic apoptosis by ABT-737. Mechanistically, we found that inhibition of electron transport markedly increases triglyceride levels in leukemia cells, further supporting the hypothesis that FAO provides electrons for the reduction of molecular oxygen in these cells. Additionally, we observed that biguanides promote an increase in superoxide production, a decrease in reduced glutathione (GSH) content, and enhanced Bak oligomerization at the outer mitochondrial membrane, all of which may be mediating increased sensitivity to ABT-737.

## RESULTS

### Metformin inhibits mitochondrial electron transport in leukemia cell lines and primary samples

**Figure 1:**
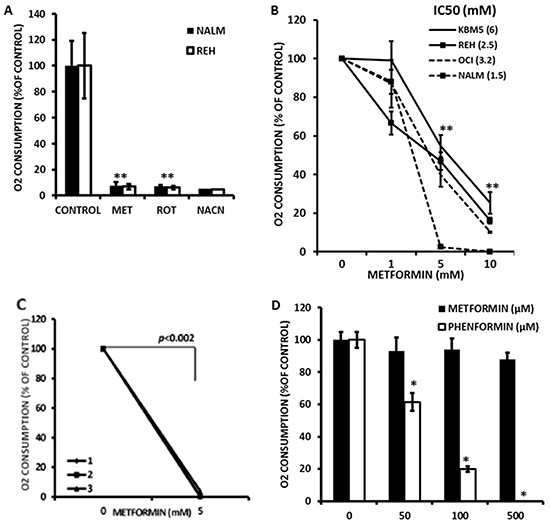
Metformin inhibits electron transport in leukemia cell lines **A.** REH and NALM-6 cells were seeded at 2×10^6^ cells/mL in 100 μl of RPMI medium in microfuge tubes and treated with 10 mmol/L Metformin (MET), 1 μmole/L rotenone or 4 mmole/L sodium cyanide for 1 hr and processed to determine oxygen consumption as described in Materials and Methods. Untreated cells were used as controls. * = *p* < 0.05 compared to control. **B.** KBM5, OCI-AML3, NALM-6, and REH cells (2×10^6^ cells/mL in microfuge tubes) were exposed to increasing concentrations (0, 1, 5, and 10 mmol/L) of Metformin for 1 hr and oxygen consumption determined as above. IC50 values for Metformin are indicated next to each cell line. ** = *p* < 0.05 compared to 0 mmole/L Metformin for all lines tested. **C.** Three primary samples (#1, #2, and #3) were seeded in 100 μl of RPMI medium in microfuge tubes and treated with 0, 5, or 10 mmol/L Metformin for 1 h and processed to determine oxygen consumption as above. Sample #2 was stained with anti-CD34 APC prior to fixation, and results are derived from CD34-positive leukemia blasts. **D.** OCI-AML3 cells were treated with 4 mmole/L sodium cyanide (as a control for inhibition of oxygen consumption) or increasing micromolar concentrations of Metformin or Phenformin for 1 hr and oxygen consumption determined as above. * = *p* < 0.05 compared to control. The data was normalized by substracting the MFI values of OCI-AML3 cells treated with 4 mmole/L sodium cyanide.

While previous studies have suggested that Metformin inhibits the molecular reduction of oxygen in hepatocytes and leukemia cell lines [14, 22], it remains to be determined if this is also true in primary leukemia blasts. To test this, we developed a novel flow cytometric method that employs the oxygen sensitive probe pimonidazole. We reasoned that if we limited air exchange by covering cell cultures with mineral oil, oxygen consumption would result in cellular hypoxia which could then be quantitated by measuring pimonidazole adducts by immunofluorescence flow cytometry. Initially, NALM-6 and REH leukemic cell lines were exposed to 10 mmole/L Metformin, 1 μmole/L rotenone or 4 mmole/L sodium cyanide for 1 hr, followed by addition of 100 μmole/L pimonidazole and processed as described in Materials and Methods. As shown in Figure 1A, a short (1 hr) exposure to Metformin inhibited the accumulation of pimonidazole adducts (*p<0.05*) in both REH and NALM-6 cells to a similar degree as rotenone or sodium cyanide (>90%), demonstrating that our methodology is adequate to monitor the effects of this biguanide on the molecular reduction of oxygen in leukemia cell lines. To further investigate the pharmacology of this effect, KBM5, OCI-AML3, NALM-6, and REH cells were exposed to increasing concentrations (1, 5, and 10 mmol/L) of Metformin for 1 hr and oxygen consumption determined as above. The results (Figure 1B) demonstrate that Metformin rapidly inhibits oxygen consumption, displaying IC50 values ranging from 1.5 (NALM-6) to 6 mmole/L (KBM5). Similar observations were made using OxygenBiosensor plates as previously reported [6] (Supplementary Figure S1). Moreover, we provide evidence that Metformin also inhibits the molecular reduction of oxygen in 3 primary leukemia samples (Figure 1C; gating in sample #2 is on CD34 (+) blasts), suggesting that this biguanide can indeed inhibit mitochondrial oxidative metabolism in primary leukemia cells. Lastly, since milli-molar concentrations of Metformin may not be achievable *in vivo*, we investigated the effects of micromolar concentrations of this agent and the related biguanide Phenformin on oxygen consumption in leukemia cells. As shown in Figure 1D, although Metformin significantly inhibited oxygen consumption at 500 μmoles/L, Phenformin was 10-fold more potent, achieving ~40% inhibition at doses as low as 50 μmoles/L. Taken together, the above results demonstrate that biguanides inhibit molecular reduction of oxygen in leukemia cells.

### Inhibition of electron transport by metformin is associated with accumulation of triglycerides

**Figure 2:**
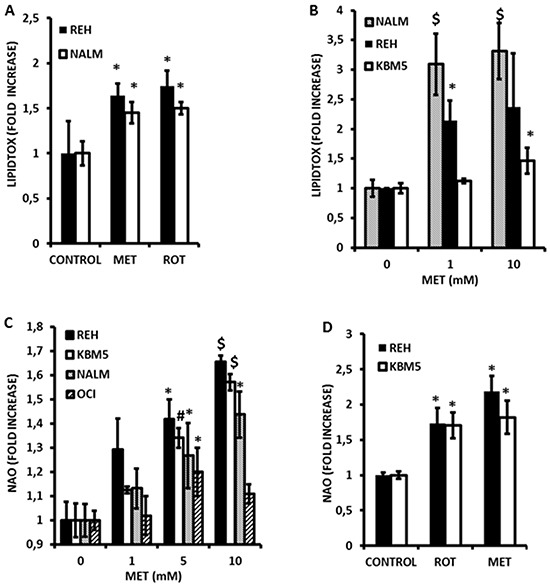
Metformin induces accumulation of triglycerides and promotes alterations in mitochondrial phospholipid content in leukemia cell lines **A.** REH and NALM-6 cells were seeded at 2×10^5 cells/mL (300 μl in 48-well plates) and treated with 10 mmol/L Metformin (MET) or 1 μmole/L rotenone for 16 hr and processed to determine neutral lipid content as described in Materials and Methods. Untreated cells were used as controls. **B.** KBM5, NALM-6, and REH cells (2×10^5 cells/mL in 48-well plates) were exposed to increasing concentrations (0, 1, and 10 mmol/L) of Metformin for 4 hr and accumulation of neutral lipids determined as above. **C.** KBM5, OCI-AML3, NALM-6, and REH cells (2×10^5 cells/mL in 48-well plates) were exposed to increasing concentrations of Metformin as above, and mitochondrial phospholipid content assessed via NAO staining as described in Materials and Methods. **D.** REH and NALM-6 cells (2×10^5 cells/mL in 48-well plates) were seeded as above and treated with Metformin (10 mmol/L) or rotenone (1 μmol/L) for 6 h and mitochondrial phospholipid content determined as above. * = *p* < 0.05 when compared to control; # = *p* < 0.005 when compared to control; $ = *p* < 0.0005 when compared to control.

We previously demonstrated that electron transport in leukemia cells is largely dependent upon FAO [7]. We therefore hypothesized that inhibition of electron transport would promote accumulation of triglycerides in these cells. To test this we treated NALM-6 and REH cells with Metformin (10 mmole/L) and rotenone (1 μmole/L) for 16 h and monitored accumulation of triglycerides via flow cytometry using the neutral lipid sensitive stain LipidTox. As shown in Figure 2A, Metformin and rotenone both significantly (*p<0.05*) increased neutral lipids in both cell lines. Additionally, Metformin promoted dose-dependent accumulation of neutral lipids as early as 4 h after treatment in KBM5, NALM-6 and REH cell lines (Figure 2B). However, significant accumulation of neutral lipids in OCI-AML3 cells was not detected at 4 h or 16 h (data not shown); perhaps indicating that Metformin also activates lipolysis in this cell line. Lastly, using the Seahorse XF96 Analyzer it was observed that metformin partially inhibited the oxidation of palmitate in KBM5 and REH cells (Supplementary Figure S5 and S6). Taken together, these observations support the notion that inhibition of electron transport in leukemia cells may antagonize FAO.

### Metformin causes alterations in mitochondrial mass and promotes superoxide generation in leukemia cells

**Figure 3:**
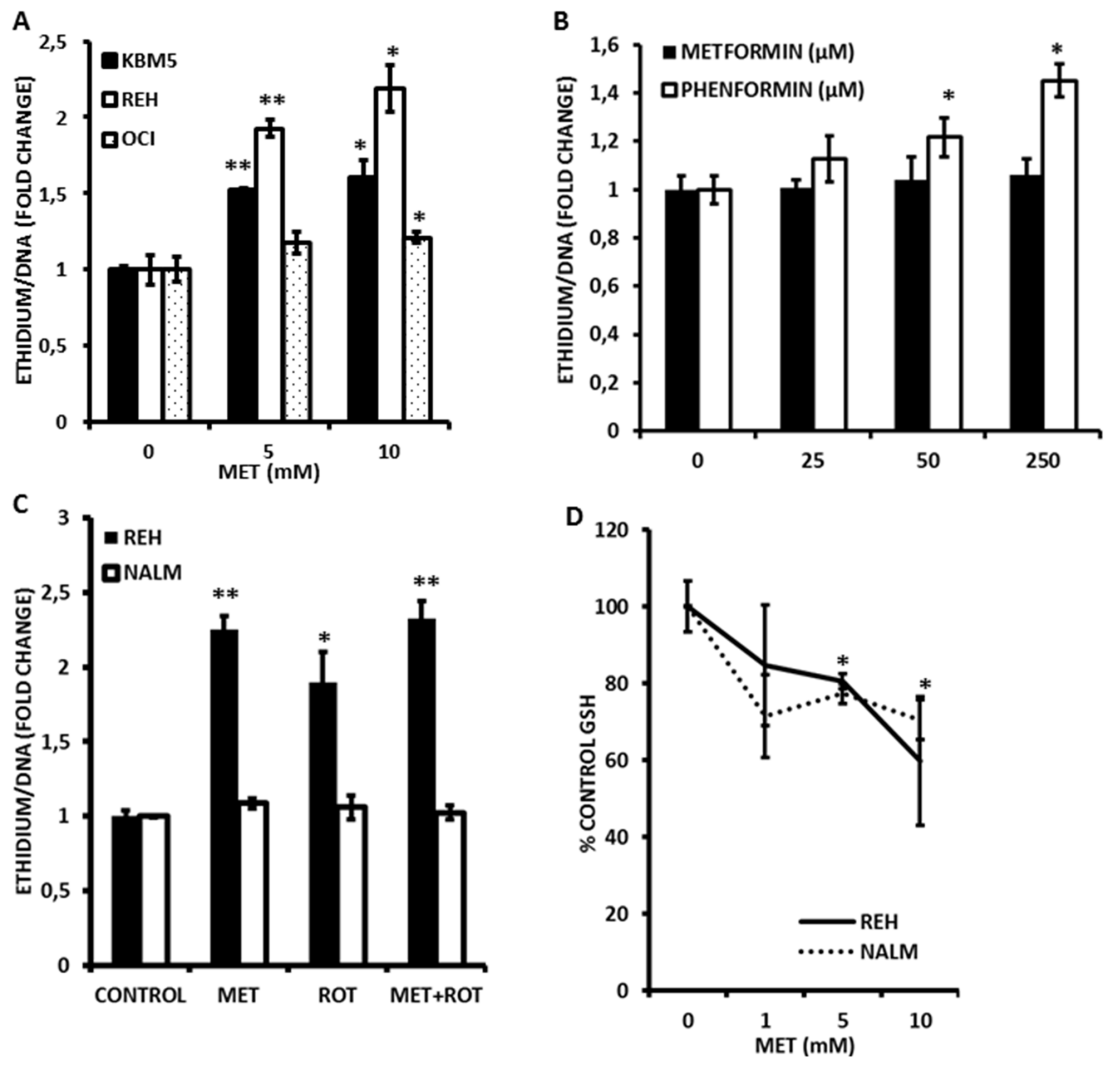
Metformin promotes superoxide generation and loss of reduced glutathione (GSH) in leukemia cells **A.** KBM5, REH, and OCI-AML3 cells were seeded at 2×10^5 cells/mL (300 μl in 48-well plates) and treated with Metformin (MET; 5 or 10 mmol/L) for 2 h and superoxide generation determined via DHE staining as described in Materials and Methods. **B.** OCI-AML3 cells were seeded at 2×10^5^ cells/mL (500 μl in 24-well plates) and treated with increasing micromolar concentrations of Metformin or Phenformin for 2 h and superoxide generation determined via DHE staining as above. **C.** REH and NALM-6 cells (2×10^5^ cells/mL in 48-well plates) were exposed to 10 mmol/L Metformin (MET) or 1 μmole/L rotenone for 2 h and superoxide generation assessed as above. **D.** REH and NALM-6 cells (2×10^5^ cells/mL in 48-well plates) were exposed to increasing concentrations (0, 1, 5, and 10 mmol/L) of Metformin for 2 h and GSH levels determined by flow cytometry as described in Materials and Methods. * = *p* < 0.05 when compared to control; ** = *p* < 0.005 when compared to control.

Since inhibition of FAO would also be predicted to increase flux of fatty acids into phospholipid synthesis, we questioned whether mitochondrial lipid mass would be altered in response to Metformin. As shown in Figure 2C, exposure to Metformin for 4 h significantly and dose-dependently increased mitochondrial mass in leukemia cell lines as monitored by flow cytometry using the fluorescent dye nonyl-acridine orange (NAO). Furthermore, rotenone similarly increased NAO accumulation (Figure 2D) supporting the notion that inhibition of electron transport is associated with structural changes in the mitochondrial membranes of leukemia cells. In addition, because fatty acid accumulation has been shown to increase production of superoxide anions [23, 24], and because Metformin inhibits complex I of the respiratory chain (a major site of superoxide production when inhibited) [16], we asked if Metformin could promote reactive oxygen species (ROS) generation in leukemia cells. As shown in Figure 3A, Metformin significantly increased superoxide production in OCI-AML3, REH, and KBM5 cells. Moreover, Phenformin was again more potent than Metformin in inducing superoxide production from OCI-AML3 cells, inducing significant increases in this ROS at doses as low as 50 μmoles/L (Figure 3B). Notably, NALM-6 cells were resistant to the pro-oxidant effects of both rotenone and Metformin (Figure 3C), suggesting that mitochondria in these cells do not produce superoxide in response to complex I inhibition and/or fatty acid accumulation, or perhaps that their superoxide dismutase activity is exceptionally high. Lastly, since superoxide production would be predicted to diminish GSH levels, and because Metformin has been reported to antagonize one-carbon metabolism, which is required for the *de novo* synthesis of GSH [25], we explored if this biguanide would reduce GSH levels in leukemia cells. Interestingly, As shown in Figure 3D, we found that Metformin significantly (*p* < 0.05 for 5 and 10 mmol/L as compared to control) reduced GSH levels by as much as 40% in REH cells, which augment superoxide production in response to Metformin, as well as in NALM-6 cells (*p* < 0.05 for 5 and 10 mmol/L as compared to control; 30% reduction at 10 mmol/L) which do not, suggesting that in addition to its pro-oxidant activity, Metformin may also antagonize one-carbon metabolism to reduce GSH pools in leukemia cells (Figure 3D). Rotenone similarly decreased GSH levels in leukemia cells lines (not shown). Together, the above data support the notion that Metformin promotes rapid increases in mitochondrial lipid mass, increases the generation of superoxide, and diminishes GSH levels.

### Metformin, phenformin, and rotenone potentiate the cytotoxic effects of the bcl-2 inhibitor ABT-737

**Figure 4:**
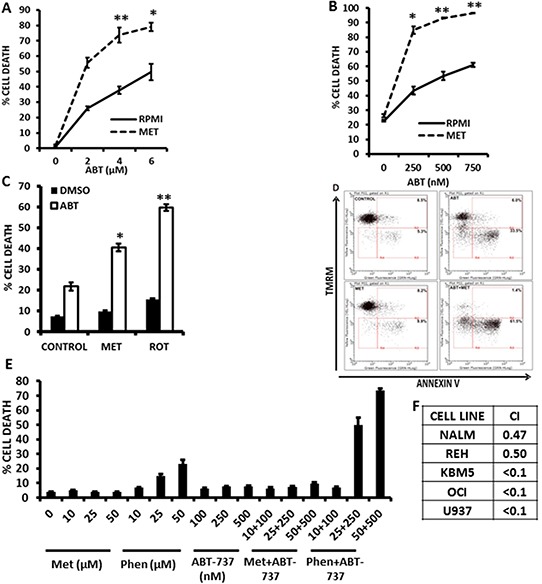
Metformin and rotenone potentiate the cytotoxic effects of the bcl-2 inhibitor ABT-737 **A.** KBM5 cells (2×10^5 cells/mL in 48-well plates) were exposed to increasing concentrations of ABT-737 (0, 2, 4, and 6 μmol/L), alone or in the presence of 10 mmol/L Metformin for 16 h and % cell death was determined via TMRM staining as described in Materials and Methods. **B.** REH cells (2×10^5 cells/mL in 48-well plates) were exposed to increasing concentrations of ABT-737 (0, 250, 500, and 750 nmol/L), alone or in the presence of 10 mmol/L Metformin for 16 h and % cell death was determined as above. **C.** REH cells seeded as above were treated with 250 nmol/L ABT-737, alone or in the presence of 10 mmol/L Metformin or 1 μmol/L rotenone for 16 h and cell death was determined as above. **D.** KBM5 cells (2×10^5^ cells/mL in 48-well plates) were exposed to 750 nmol/L ABT-737, alone or in the presence of 15 mmol/L Metformin and Annexin staining was quantitated as described in Materials and Methods. **E.** OCI-AML3 cells were seeded at 2×10^5^ cells/mL (500 μl in 24-well plates) and treated with increasing micromolar concentrations of Metformin or Phenformin, ABT-737, or combinations of biguanides and ABT-737 in an isobologram design. Viable cells were determined by propidium iodide staining and flow cytometry as described in Materials and Methods. * = *p* < 0.05 when compared to ABT-737 alone; ** = *p* < 0.005 when compared to ABT-737 alone. **F.** Leukemia cells (2×10^5^ cells/mL in 48-well plates) were treated with Metformin (5, 10, or 15 mmol/L) or ABT-737 (250, 500, 750 nmol/L for REH and KBM5; 1, 2, 3 μmol/L for OCI-AML3; 1, 2, 3 μmol/L for NALM-6; and 250, 500, and 750 nmole/L for U937), and the fixed ratio combinations of Metformin + ABT-737 for 16 h and cell death was determined as described in Materials and Methods. Isobologram analysis and CI were determined using Calcusyn version 2.1. Averaged CI values are shown for each cell line.

**Figure 5:**
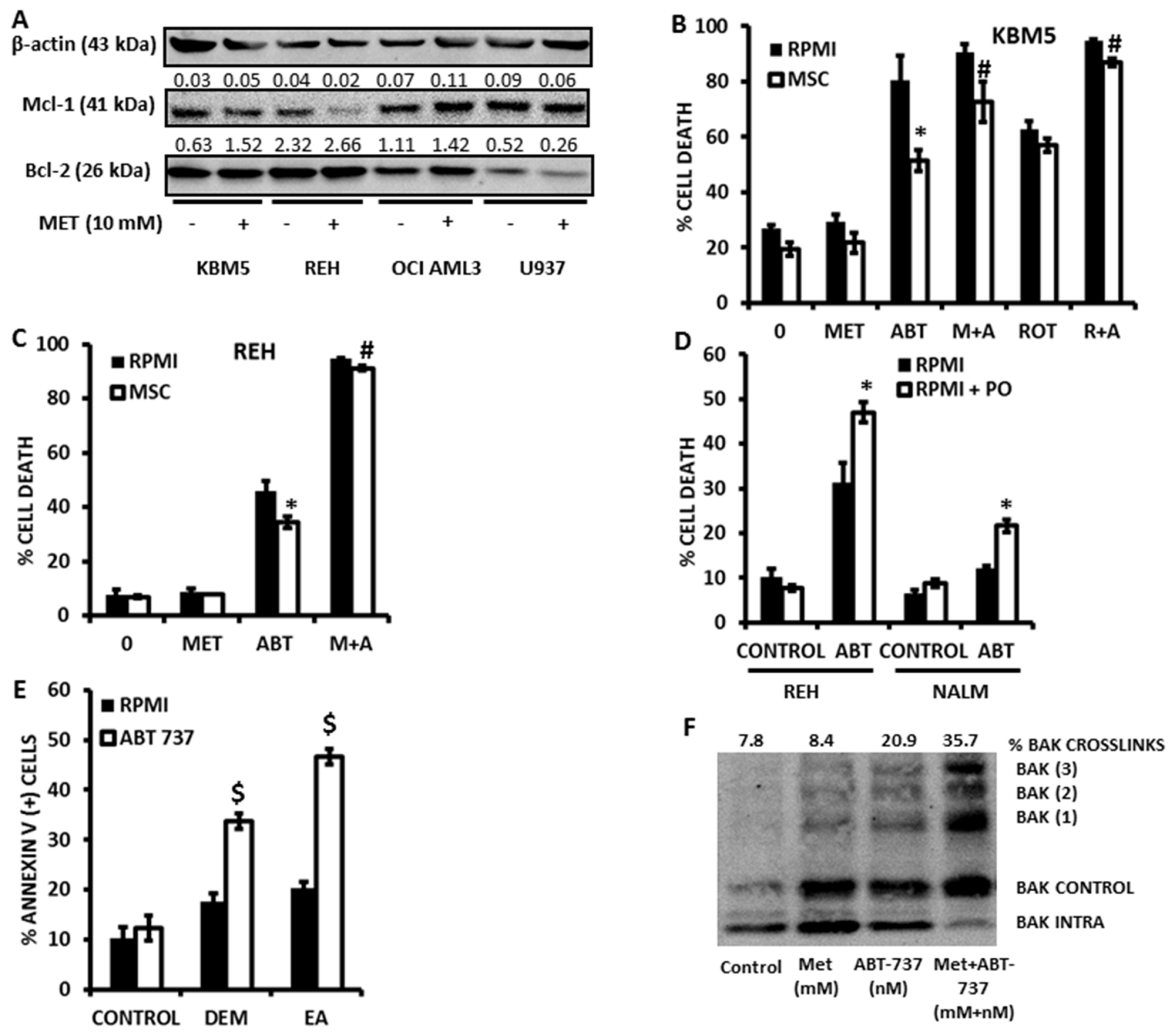
Metformin overcomes the protective effects of bone marrow derived MSC **A.** KBM5, REH, OCI AML3, U937 (0.2×10^6 cells/ml) were seeded in T25 flasks and treated or not with Metformin (10 mM) for 16 hrs. After incubation, the cells were counted and lysed to perform immunoblot detection of Bcl-2 and Mcl-1 proteins. **B.** KBM5 cells were cultured alone (2×10^5 cells/mL in 300 μl in 48-well plates), or on a feeder layer of 1×10^4 bone marrow derived MSC, and exposed to 4 μmol/L ABT-737 +/− 10 mmol/L Metformin or 1 μmol/L rotenone. Cells were incubated for 16 h and % cell death was determined as described in the Materials and Methods in the CD90 (−) compartment. **C.** REH cells were cultured alone (2×105 cells/mL in 300 μl in 48-well plates), or on a feeder layer of 1×104 bone marrow derived MSC, and exposed to 500 nmol/L ABT-737 +/− of 10 mmol/L Metformin. % cell death was determined as above. * = *p* < 0.05 when compared to cells cultured alone; # = *p* < 0.05 when compared to ABT-737 alone in coculture. **D.** REH and NALM-6 cells (2×10^5 cells/mL in 48-well plates) were cultured in RPMI or RPMI supplemented with 200 μmol/L palmitoleate (PO), and treated with ABT-737 (500 nmol/L for REH and 4 μmol/L for NALM-6) for 16 h and cell death determined by flow cytometry as described in Materials and Methods. * = *p* < 0.01 when compared to RPMI without PO. **E.** OCI-AML3 cells (2×10^5 cells/mL in 48-well plates) were treated with 250 nmol/L ABT-737, +/− 200 μmol/L ethacrynic acid (EA) or 2 mmol/L diethylmaleate (DEM) for 16 h and apoptosis was determined by Annexin V staining as described in Materials and Methods. $ = *p* < 0.0005 when compared to ABT-737 alone. **F.** U937 cells were seeded at 2×10^5^ cells/mL (15 mls in T-75 culture flasks) and treated with 500 nmol/L ABT-737 in the presence or absence of 10 mmol/L Metformin for 4 h. Bak crosslinking was determined as described in Materials and Methods.

Since we previously reported that pharmacological inhibition of FAO sensitizes leukemic cells to cell death induced by the bcl-2 inhibitor ABT-737 [7], and since GSH has been shown to antagonize the cytotoxicity of ABT-737 [26], we hypothesized that Metformin, Phenformin, and rotenone – via blockage of electron transport and subsequent inhibition of FAO, and/or reduction in antioxidant capacity – would also potentiate the cytotoxic effects of ABT-737. As shown in Figure 4A and 4B, exposure of KBM5 and REH cells to Metformin reduced the IC50 for ABT-737 by 75% and 92%, respectively. Rotenone similarly potentiated the effects of ABT-737 at inducing cell death in leukemia cells (Figure 4C). Furthermore, the reduction in the IC50 dose was mediated by increased apoptosis, as assessed by Annexin V staining in KBM5 cells (Figure 4D). Notably, low micromolar doses of Phenformin, but not Metformin, synergized with ABT-737 in inducing cytotoxicity in OCI-AML3 cells (Figures 4E and 4F; average combination index (CI) = 0.15), demonstrating the higher potency of this biguanide. Nevertheless, millimolar concentrations of Metformin synergized with ABT-737 to induce cell death in various leukemia cell lines (Figure 4F, and Supplementary Figures S2-S4). To determine if the sensitivity to ABT 737 and Metformin treated cells was mediated by alterations in Bcl-2 or Mcl-1 protein levels, we treated KBM5, REH, OCI-AML3 and U937 cells with Metformin (10 mmoles/L) for 16 hrs, and evaluated Bcl-2 and Mcl-1 expression by immunoblot. Interestingly, Mcl-1 levels were decreased only in REH cells, whereas Bcl-2 levels were unchanged in all cell lines after metformin treatment (Figure 5A), suggesting that alterations in antiapoptotic proteins may not be an absolute requirement for the observed synergy between Metformin and ABT-737. Additionally, immunoblot experiments also revealed that metformin, alone or in combination with ABT-737 did not elicit phosphorylation of AMPK at threonine 172 (Supplementary Figure S7), suggesting that the observed effects of metformin in leukemia cells are likely AMPK-independent.

Notably, although KBM5 or REH cells cocultured with bone marrow-derived MSC were significantly (*p<0.01*) protected from the cytotoxic effects of ABT-737, both rotenone and Metformin antagonized this protective effect of MSC (Figures 5B and 5C), indicating that, like previously reported for inhibition of FAO [7], inhibition of electron transport can overcome the chemoprotective effects of the leukemic microenvironment. Addition of palmitoleate – a reported cytoprotective monounsaturated fatty acid [27] – also sensitized leukemia cells to the cytotoxicity of ABT-737 (Figure 5D); substantiating the idea that accumulation of intracellular fatty acids may be associated with the sensitizing effects of Metformin. Moreover, as shown in Figure 5E, addition of diethylmaleate (DEM) or ethacrynic acid (EA) also sensitized OCI-AML3 cells to the cytotoxic effects of ABT-737, suggesting that the observed reduction in GSH levels induced by Metformin may also contribute to the synergistic interaction of this biguanide with ABT-737. Mechanistically, as previously reported for etomoxir [7], Metformin potentiated oligomerization of Bak in leukemia cells treated with ABT-737 (Figure 5F), suggesting that the observed synergy is mediated, at least in part, by enhanced outer mitochondrial membrane permeabilization. Taken together, the above data support the hypothesis that pharmacological inhibition of electron transport and the subsequent accumulation of intracellular fatty acids and/or reduction in GSH levels as a result of exposure to Metformin sensitizes leukemia cells – cultured alone or on bone marrow-derived MSC feeder layers – to apoptosis induction by ABT-737.

### Metformin and phenformin potentiate the cytotoxicity of ABT-737 in primary leukemia samples

**Figure 6:**
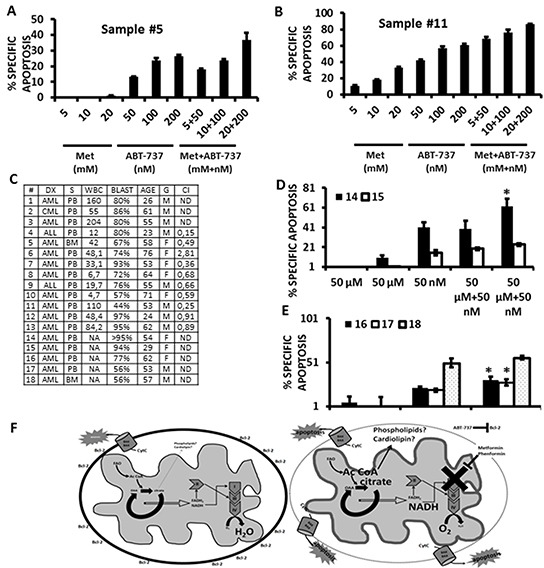
Metformin and Phenformin potentiate the cytotoxicity of ABT-737 in primary leukemia CD34 (+) cells **A.** and **B.** Two AML primary samples were exposed to increasing concentrations of Metformin, ABT-737, or a combination of each agent using a 1:1 fixed dose increase schedule. Apoptosis was monitored quantitated by flow cytometry, gating on CD34 (+) cells as described in Materials and Methods. **C.** Summary of CI values (10 samples) and patient characteristics for the primary leukemia samples used in this study. Acute lymphoblastic leukemia samples were analyzed by gating on leukemic blasts by FSC/SSC. **D.** Two primary AML samples were exposed to micromolar concentrations (50 μmoles/L) of Metformin or Phenformin, +/− 50 nmoles/L ABT-737 for 24 h and apoptosis quantitated by flow cytometry as above. * = p<0.05 from ABT-737 alone. **E.** 3 primary AML samples were exposed to 20 μmoles/L Phenformin, +/− 20 nmles/L ABT-737, and apoptosis determined by flow cytometry, gating on CD34 (+) cells as in *A* and *B*. * = p<0.05 from ABT-737 alone. **F.** Left – untreated leukemia cell; Right – biguanides inhibit electron transport leading to the accumulation of citrate which may promote *de novo* phospholipid/cardiolipin synthesis and changes to the topology of the inner mitochondrial membrane (represented by the thicker grey line). These changes potentiate the permeabilization of the outer mitochondrial membrane (represented by the dotted line) and release of apoptogenic factors induced by ABT-737.

To investigate if the observed synergy of biguanides with ABT-737 could also be observed with primary leukemic cells, we exposed 2 primary acute myelogenous leukemia samples to increasing concentrations of Metformin, ABT-737, or the combination of these two agents using a fixed dosing schedule for 24 h, and analyzed viable CD34(+) cells by flow cytometry. As shown in Figures 6A and 6B, Metformin and ABT-737 synergized (averaged CI <0.8) to induce apoptosis in primary CD34 (+) AML cells. Additional testing of 8 more AML or acute lymphoblastic leukemia (ALL) samples demonstrated that the combination of ABT-737 with Metformin resulted in synergic CIs in ~80% of primary leukemia samples (summarized in Figure 6C). We also compared the effects of micromolar doses of Metformin or Phenformin, alone or in combination with ABT-737 (50 nmoles/L) on the apoptosis of 5 additional primary samples, and observed that at these low micromolar doses only Phenformin significantly (p<0.05) sensitized 3 out of 5 primary leukemic cells to cell death induced by ABT-737 (Figures 6D and 6E). Lastly, we exposed normal CD34 (+) bone marrow derived cells to increasing doses of ABT-737 in combination with increasing doses of metformin, and found that despite evidence of toxicity from ABT-737, metformin did not potentiate this effect even at the highest (20 mmoles/L) dose tested (Supplementary Figure S8). Taken together, the above results support the notion that biguanides can potentiate the toxicity of ABT-737 in primary leukemic samples, but not in normal hematopoietic progenitors.

## DISCUSSION

Although we had shown previously that FAO is the predominant electron source for the molecular reduction of oxygen and promotes resistance to apoptosis induction in leukemic cells [7], it was not known if mitochondrial electron transport *per se* antagonizes apoptosis in leukemic cells. In addition, while our previous work supports the use of etomoxir as a FAO inhibitor and a chemosensitizing agent, the use of this agent is limited by its lack of FDA approval, its long term toxicity [28, 29], and its high cost, prompting us to search for alternative candidates that may be more clinically relevant. It has been previously demonstrated that Metformin, a relatively non-toxic biguanide widely used by millions of type II diabetics around the world, possesses antileukemic activity via, in part, AMPK-mediated antagonism of mTOR/Akt signaling [30]. Nonetheless, AMPK-independent antitumor mechanisms have also been ascribed to Metformin [18–22], and intriguing recent evidence suggests that the antidiabetic effects of Metformin may be mediated solely by AMPK-independent inhibition of electron transport in hepatocytes [14], which tempted us to question if this agent could indirectly inhibit FAO in leukemia cells via accumulation of NADH. Could Metformin be a suitable non-toxic alternative to etomoxir?

Herein we report that Metformin, and the related biguanide, Phenformin, rapidly and profoundly inhibit oxygen consumption in leukemia cells, and that, as predicted for an inhibitor of mitochondrial complex I, this effect is associated with increased superoxide generation. We also show that accumulation of intracellular triglycerides closely follows the inhibition of oxygen consumption by Metformin or rotenone, lending support to the notion that inhibition of electron transport and the subsequent accumulation of NADH may antagonize FAO, and redirect FAO substrates to neutral lipid stores. Notably, in p53-wild-type OCI-AML3 cells we do not detect accumulation of triglycerides, and we speculate that perhaps in agreement with previous observations,p53 activity accompanies increased lipolysis [31]. Importantly, our results demonstrate for the first time that Metformin and Phenformin potentiate the cytotoxic effects of the bcl-2 antagonist ABT-737 in leukemia cell lines and primary samples, supporting the notion that inhibition of electron transport sensitizes leukemia cells to induction of intrinsic apoptosis.

Mechanistically, our palmitoleate experiments suggest that accumulation of free fatty acids in response to inhibition of electron transport may sensitize leukemia cells to the cytotoxic effects of ABT-737. Additionally, our results support the notion that inhibition of electron transport by Metformin or rotenone causes increases in mitochondrial phospholipid content as monitored by NAO staining. Alternatively, in light of a report by Garcia-Fernandez et al demonstrating a higher affinity of NAO for state 3 (coupled; ADP-dependent) respiring mitochondria [32], it is conceivable that the increase in NAO staining reflects, perhaps in addition to some phospholipid accumulation, topological exposure of cardiolipin and/or other phospholipids that accompany reduced electron transport and may be indicative of respiratory coupling. Together with our finding that metformin lowers the threshold for ABT-737 induced Bak crosslinking – an obligate step in activation of intrinsic apoptosis [33, 34]– the above lend support to a paradigm in which increased exposure of cardiolipin (or other phospholipid) molecules favors interaction with caspase cleaved bid (t-bid) [35], activated Bax [36], or activated Bak [37], and facilitates the permeabilization of the outer mitochondrial membrane and subsequent release of proapoptotic proteins (Figure 6F).

Our observation that Metformin diminishes GSH levels in all 4 cell lines examined was surprising, since we had predicted this effect to be dependent on the generation of superoxide, and superoxide production in response to Metformin was not observed in NALM-6 cells, either by rotenone or Metformin. Thus, we speculate that our results are in agreement with a recent report that suggests this biguanide can antagonize one-carbon metabolism which is required for the synthesis of GSH [25], and our experiments with diethylmaleate and ethacrynic acid demonstrate that GSH depletion may also contribute to the synergic interaction of Metformin with ABT-737.

Our group has previously demonstrated that leukemia cells are exquisitely sensitive to ABT-737 [38], and its orally bioavailable derivative, ABT-199 (Venetoclax) is currently being evaluated in several clinical trials for its safety and efficacy against chronic lymphocytic leukemia [39–41]. A recent update on a Phase II study with relapsed/refractory chronic lymphoid leukemia patients with 17p deletion reports that oral administration of Venetoclax is well tolerated and is associated with a response rate of 79.4% with no detectable minimal residual disease in 20% of responders [10]. Still, any strategy that could potentiate the antileukemic effects of Venetoclax is of utmost interest. Metformin is a very attractive combination partner as its safety has been demonstrated when used alone or combined with traditional chemotherapeutic schemes in several clinical trials [42–45]. It is also tempting to speculate that the greater potency of Phenformin over that of Metformin may be more desirable in a possible combination strategy with Venetoclax, although the toxicity of Phenformin may also be limiting in this scenario. Nevertheless, our study is limited in its ability to predict therapeutic efficacy due to the lack of in vivo data. In summary, our findings suggest that pharmacologic inhibition of electron transport maximizes the antileukemic effects of ABT-737, and lend support to the use of Metformin or Phenformin in combination with Venetoclax as a potential therapeutic strategy for the treatment of leukemia.

## MATERIALS AND METHODS

### Cell lines, chemicals, and biochemical

OCI-AML3 (Human Acute Myeloid Leukemia), REH (Acute Lymphocytic leukemia, non-T; non-B), NALM-6-6 (Human B cell precursor leukemia), and KBM5 (Chronic Myeloid Leukemia) cell lines; were provided by the Section of Molecular Hematology and Therapy of the University of Texas MD Anderson Cancer Center. The cells lines were pathogen tested and authenticated through STR (Short Tandem Repeat) method on 2010.

Cells were maintained in RPMI supplemented with 10% fetal calf serum (FCS), 1% glutamine, 100 U/ml penicillin, 100 μg/ml streptomycin, and 1 mg/L amphotericin B in a 37°C incubator containing 5%CO2. MSC were derived from normal bone marrow samples obtained with informed consent in accordance with regulations and protocols approved by the Human Subjects Committee of the University of Texas M.D. Anderson Cancer Center. Anti-CD90 conjugated to APC, anti-CD34 conjugated to APC, and Annexin V conjugated to FITC were obtained from EBiosciences (San Diego, CA). LipidTox Green for neutral lipids, Tetramethyl Rhodamine Methyl Ester (TMRM), dyhydroethidine (DHE), CellTracker Green and nonyl acridine-orange (NAO) were obtained from Invitrogen (Carlsbad, CA). Rotenone, Metformin, Phenformin and sodium cyanide were from Sigma-Aldrich and dissolved in DMSO (rotenone) or water. ABT-737 was synthesized at the University of Texas MD Anderson Cancer center based on the published structure and dissolved in DMSO.

### Measurement of apoptosis and viable cell numbers by flow cytometry

After appropriate treatments, cells were washed twice in PBS and then resuspended in 100 μl Annexin binding buffer containing a 1:100 dilution of Annexin V–FITC and 50 nmol/L tetramethyl-rhodamine methyl ester; where appropriate for MSC coculture experiments, a 1:100 dilution of anti-CD90 APC-conjugated antibody was added. CD90 was used to discriminate MSC (positive) from leukemia cells (negative). In some experiments viable leukemia cells were determined by staining with propidium iodide (Sigma), and viable leukemia progenitors were assessed after staining with anti-CD34 APC-conjugated antibody. Cells were then analyzed by flow cytometry in a Guava EasyCyte 6-2L capillary cytometer (Merck, Millipore) or a FACSCalibur cytometer (BD Biosciences) using a 488-nm argon ion and 633-nmHeNe excitation lasers.

### Measurement of oxygen consumption by flow cytometry

Briefly, cells were seeded (2.0 × 10^^6^/ml) in complete RPMI medium in microfuge (1.5 mL) tubes and treated with sodium cyanide (5 mM) for 1hr, or rotenone or Metformin at the indicated doses for 2 h at 37°C, 5% CO2. Untreated samples served as controls. Pimonidazole (Chemicon International, Temecula, CA) was added (100 μmol/L) to all samples, except a control (untreated) sample which served to assess background staining. Immediately after the addition of pimonidazole, cell suspensions were covered with 0.5 ml of mineral oil and further incubated for 2 h at 37°C. After incubation, a micropipetor was inserted through the mineral oil, taking care to expel any trapped oil, and the cells were recovered slowly, being careful not to collect any oil. The cells were centrifuged (1200 *g*, 5 min), washed once in PBS, resuspended in 250 μl of 1.6% formaldehyde (in PBS), and incubated at 23°C for 15 min. After fixation, the cells were centrifuged (1200 *g*, 5 min), resuspended in 1 ml of 100% methanol, and incubated at −20°C for 1 h to overnight. Cells were again centrifuged (1200 *g*, 5 min), washed twice in PBS supplemented with 2% FCS, and stained for 1 hr at 23°C with 100 – 1,000 μl of a 1:200 - 1:500 dilution of anti-pimonidazole antibody (Chemicon International, Temecula, CA) in PBS supplemented with 2% FCS, 0.002% sodium azide. Stained cells were washed twice in PBS and fluorescent emission at 525 nm analyzed by flow cytometry in a Guava EasyCyte 6-2L capillary cytometer or a FACSCalibur cytometer using a 488-nm argon ion excitation laser. Results are expressed as mean fluorescent intensity (MFI).

### Measurement of superoxide production

After appropriate treatments, cells were loaded with DHE (500 nmol/L) for 30 min in a 37°C incubator containing 5%CO2, followed by addition, or not, of 1 μmol/L rotenone, and a further incubation for 30 min. Cells were then washed twice in PBS, and fluorescent emission at 583 nm analyzed by flow cytometry in a Guava EasyCyte 6-2L capillary cytometer or a FACSCalibur cytometer using a 488-nm argon ion excitation laser. Results are expressed as MFI.

### Measurement of neutral lipid content

After appropriate treatments, cells were loaded with LipidTox neutral green stain for 45 min in a 37°C incubator containing 5%CO2, followed by two washes in PBS. Fluorescent emission at 525 nm was analyzed by flow cytometry in a Guava EasyCyte 6-2L capillary cytometer (Merck, Millipore) using a 488-nm argon ion excitation laser. Results are expressed as MFI.

### Measurement of mitochondrial phospholipid mass

After appropriate treatments, cells were loaded with nonyl-acridine-orange (NAO; 100 nmole/L) for 30 min in a 37°C incubator containing 5%CO2, followed by two washes in PBS. Fluorescent emission at 525 nm was analyzed by flow cytometry in a Guava EasyCyte 6-2L capillary cytometer (Merck, Millipore) using a 488-nm argon ion excitation laser. Results are expressed as MFI.

### Measurement of reduced glutathione (GSH) content

After appropriate treatments, cells were loaded with CellTracker Green (200 μmole/L) for 30 min on ice, followed by two washes in cold PBS. Fluorescent emission at 525 nm was analyzed by flow cytometry in a Guava EasyCyte 6-2L capillary cytometer (Merck, Millipore) using a 488-nm argon ion excitation laser. Results are expressed as MFI.

### Bak crosslinks

Bak crosslinks were investigated as previously described [7]. Briefly, after exposure of U937 cells to ABT-737 and/or Metformin, mitochondrial extracts generated by hypotonic lysis were resuspended in 150 mM NaCl, 10 mM HEPES (pH 7.4),and 1% CHAPS at 1 mg/ml of protein and treated with 0.4 mM bismaleimidohexane (Thermo Scientific) for 1 h at 23°C. Lysates (12.5 μg of protein per well) were then subjected to SDS-polyacrylamide gel electrophoresis in 12% polyacrylamide gels followed by protein transfer to polyvinyl difluoride (PVDF) membranes (Thermo Scientific) and immunoblotted with Bak antibody (clone D2D3 Rabbit mAb, Cell Signaling Technology). Signals were detected by immunofluorescence, using fluorochromes conjugated anti-rabbit secondary antibodies and the Odyssey Imaging System (Li-Cor Biosciences, Lincoln, NE, USA).

### Immunoblotting

After appropriate treatments cells were collected, counted and 1 million of cells were re-suspended in 2X Laemmli Buffer (4% SDS, 20% Glycerol, 10% 2-Mercaptoethanol, 0.004% Bromophenol Blue and 0.125M Tris HCL). Twenty microliter of protein were then subjected to SDS-polyacrylamide gel electrophoresis in 12% polyacrylamide gels followed by protein transfer to polyvinyl difluoride (PVDF) membranes (Thermo Scientific) and immunoblotted with mouse monoclonal anti Bcl2 antibody (Dako, Carpentaria, CA), rabbit monoclonal anti Mcl-1 antibody, rabbit monoclonal anti-AMPKα, rabbit monoclonal anti-phospho(Thr172)AMPKα (All from Cell Signaling Technology), and mouse monoclonal anti-β-actin (Sigma-Aldrich). Signals were detected by immunofluorescence, using fluorochromes conjugated anti-rabbit and anti-mouse secondary antibodies and the Odyssey Imaging System (Li-Cor Biosciences, Lincoln, NE, USA).

### Primary samples

Bone marrow or peripheral blood samples were obtained for in vitro studies from patients with AML. Some samples were collected during routine diagnostic procedures after informed consent, and within 2 h after sample collection, mononuclear cells were separated by Ficoll-Hypaque (Sigma-Aldrich) density gradient centrifugation and used immediately. Frozen samples were obtained from the hematology cell bank at the BC Cancer Agency under protocol H12-00727. All experiments with primary samples were performed in triplicate.

### Statistics

Unless otherwise indicated, results are expressed as mean ± SD of at least 3 independent experiments. All experiments were performed in triplicate and repeated at least twice. *P* values were determined by unpaired student t-test. A *P* value less than 0.05 was considered significant.

## SUPPLEMENTARY METHODS FIGURES


