# Supplementary material for: Biguanides sensitize leukemia cells to ABT-737-induced apoptosis by inhibiting mitochondrial electron transport
Source: Oncotarget. 2016 Jun 6;7(32):51435–49. doi: 10.18632/oncotarget.9843 (PMC5239486; doi:10.18632/oncotarget.9843)
Supplement: Supplementary file 1 [file oncotarget-07-51435-s001.pdf]

# Biguanides sensitize leukemia cells to ABT-737-induced apoptosis by inhibiting mitochondrial electron transport

## SUPPLEMENTARY METHODS

### Effects of metformin and ABT-737 on normal CD34-positive cells

Cord blood samples from healthy donors were obtained by informed consent from The University of Texas MD Anderson Cancer Center according to protocols approved by the institute's Institutional Review Board. Mononuclear cells were isolated by Ficoll (Sigma-Aldrich) density centrifugation and used immediately. The isolated cells were treated with ABT-737, metformin or both in RPMI-1640 medium containing 10% fetal bovine serum for 16 hours. The primary cells were then stained with hCD34-APC, Annexin V-FITC and DAPI. The percentage of apoptosis (Annexin $V^{+}$) was measured by a Gallios flow cytometer (Beckman Coulter, Indianapolis, IN). Live stem/progenitor cells were also enumerated using CountBright beads (Life Technologies, Carlsbad, CA) after gating on CD34+ cells. Data were analyzed using Kaluza software (Beckman Coulter).

### Measurement of oxygen consumption and extracellular acidification rate by Seahorse system

KBM5 and REH cells ($0.4\times10^{\wedge 6}$ cells/ml in 10 ml) were seeded in T75 flasks during the time of treatment (2 hrs) in substrate-limited medium as suggested by the manufacturer. After incubation, the cells were spun down, re-suspended in FAO medium and seeded in XF96e Seahorse Biosciences plates at a concentration of 0.2-0.3 million of cells/well. Fifteen minutes before the initial measurement, Etomoxir (40 µM) was added to the control wells; and immediately before the start of data acquisition, substrates were added to the specific wells. Oxygen consumption (OCR) and extracellular acidification rate (ECAR) were determined per the manufacturers' instructions.

## SUPPLEMENTARY FIGURES

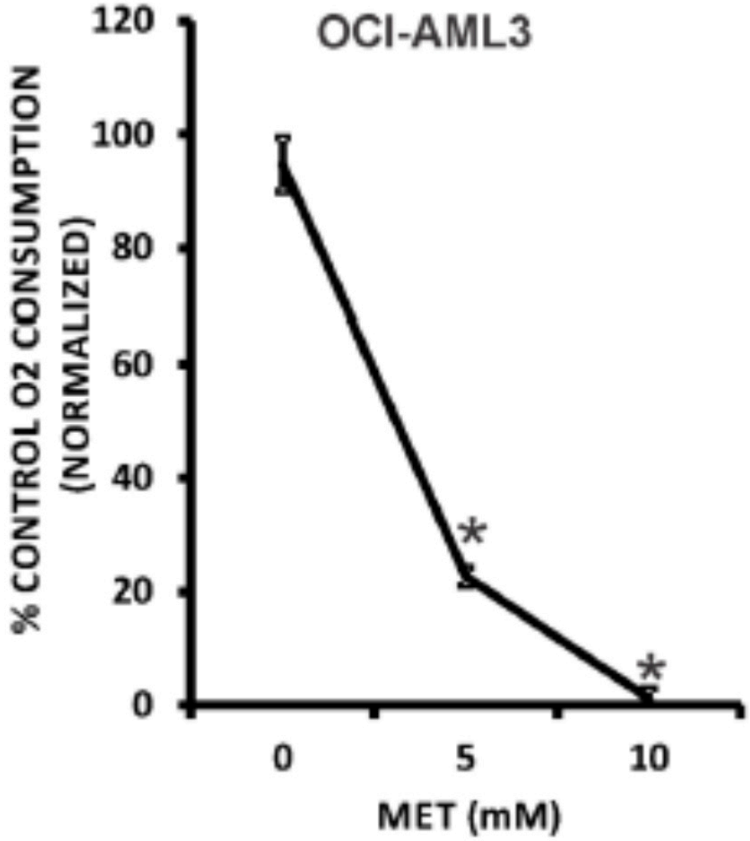


**Supplementary Figure S1: Metformin inhibits oxygen consumption in leukemia cells.** OCI-AML3 cells were seeded in 100 µl (2×10^5 cells/mL) in oxygen biosensor plates, treated with the indicated doses of Metformin or 4 mM NaCN, overlaid with mineral oil, and incubated for 4 h at 37°C. Fluorescence was read (ex 485, em 520) in a Fluostar Optima fluorescent plate reader. Fluorescent intensities were corrected for NaCN values (background substraction) and results expressed as % control. * = $p < 0.001$.

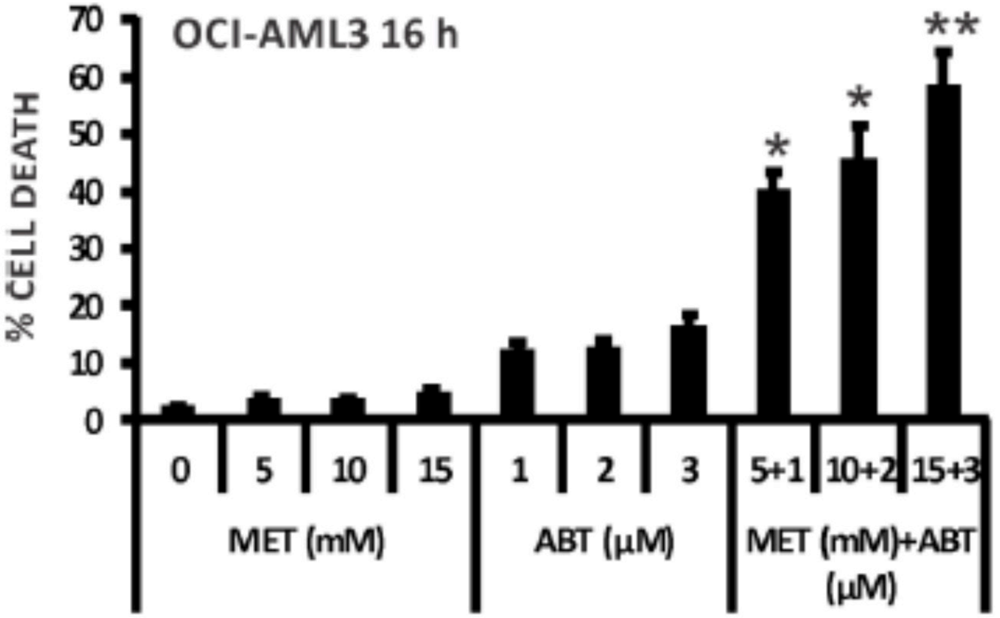


**Supplementary Figure S2: Fixed dose ratio analysis of the combination of Metformin and ABT-737 in OCI-AML3 cells.** OCI-AML3 cells (2×10^5 cells/mL in 48-well plates) were treated with the indicated doses of Metformin or ABT-737 and the fixed ratio combinations of Metformin + ABT-737 for 16 h and cell death determined as described in Materials and Methods. * = $p < 0.05$ when compared to ABT-737 alone. ** = $p < 0.01$ when compared to ABT-737 alone.

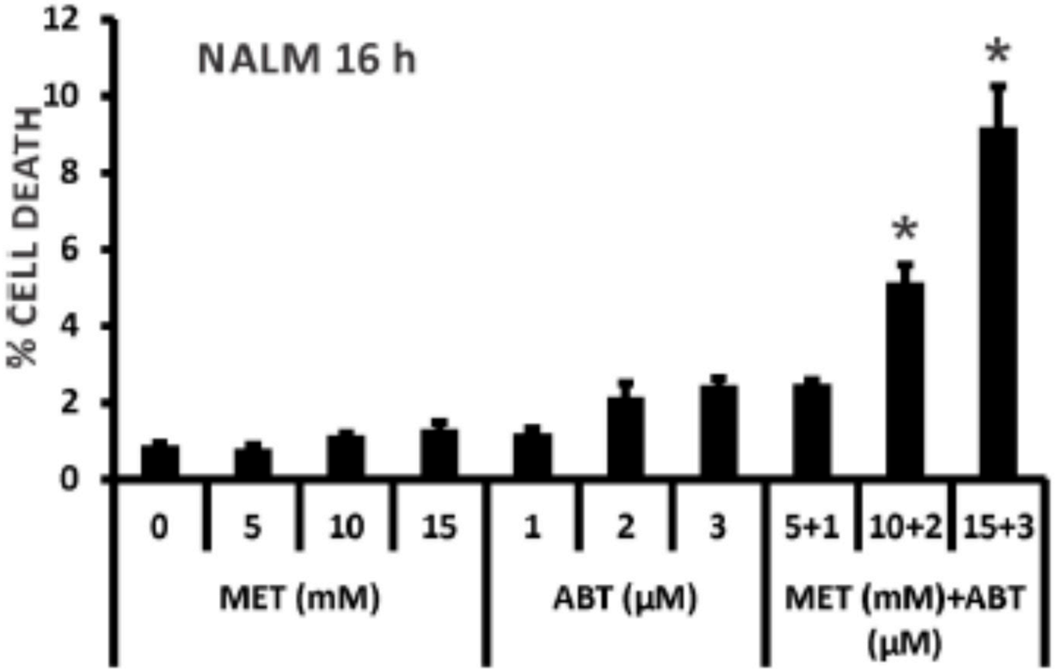


**Supplementary Figure S3: Fixed dose ratio analysis of the combination of Metformin and ABT-737 in NALM cells.** NALM cells (2×10^5 cells/mL in 48-well plates) were treated with the indicated doses of Metformin or ABT-737, and the fixed ratio combinations of Metformin + ABT-737 for 16 h and cell death determined as described in Materials and Methods. * = $p < 0.05$ when compared to ABT-737 alone.

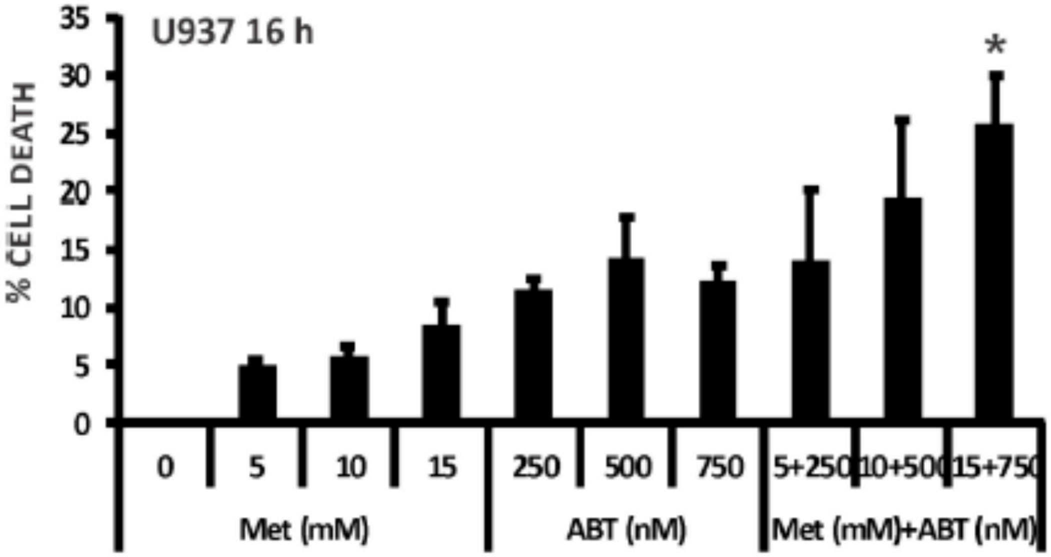


**Supplementary Figure S4: Fixed dose ratio analysis of the combination of Metformin and ABT-737 in U937 cells.** U937 cells (2×10^5 cells/mL in 48-well plates) were treated with the indicated doses of Metformin or ABT-737, and the fixed ratio combinations of Metformin + ABT-737 for 16 h and cell death determined as described in Materials and Methods. * = $p < 0.05$ when compared to ABT-737 alone.

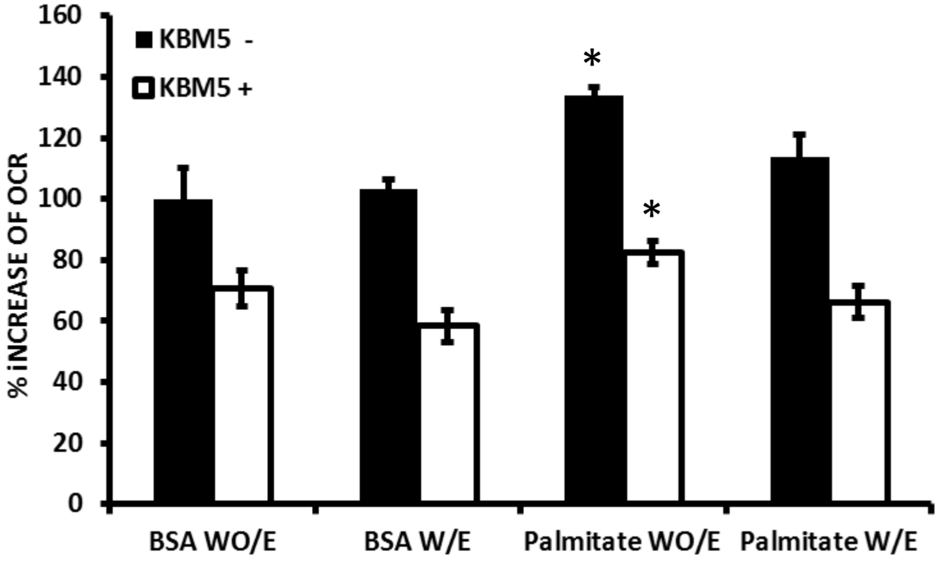


**Supplementary Figure S5: Palmitate addition recovers oxygen consumption inhibited by Metformin.** KBM5 cells (0.4×10^6 cells/ml) were seeded in T75 flasks and treated with Metformin for 2 hrs. After treatment, cells were transferred to XF96e Seahorse Biosciences plates and exposed to different substrates (Bovine Serum Albumin (BSA), Palmitate) or inhibitors (Etomoxir). Oxygen consumption (OCR) was determined and expressed as values normalized to BSA-only treated cells (BSA WO/E; defined as 100%). * = $p<0.05$ from BSA-only treated cells; ** = $p < 0.001$ from palmitate WO/E, and no metformin treatment.

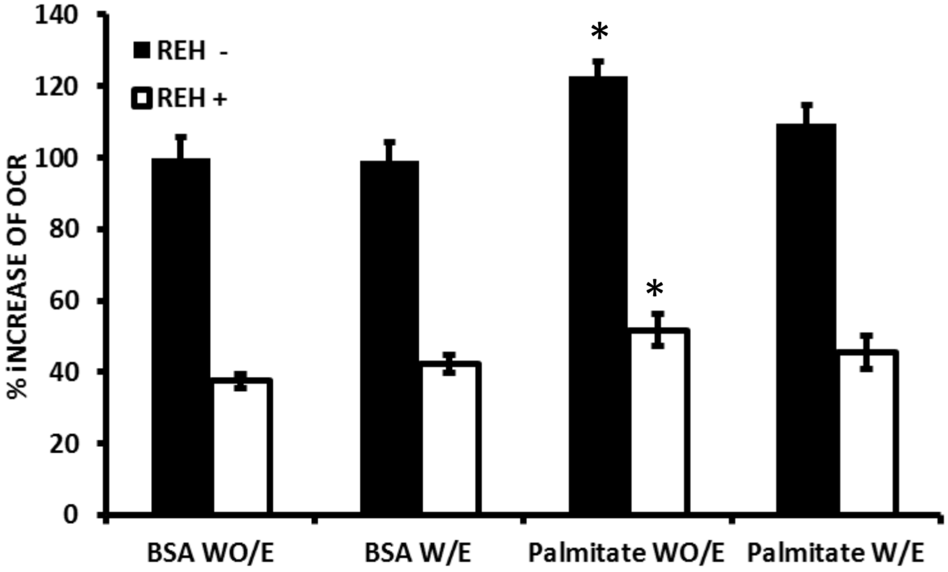


**Supplementary Figure S6: Palmitate addition recovers oxygen consumption inhibited by Metformin.** REH cells (0.4×10^6 cells/ml) were seeded in T75 flasks and treated with Metformin for 2 hrs. After treatment, cells were transferred to XF96e Seahorse Biosciences plates and exposed to different substrates (Bovine Serum Albumin (BSA), Palmitate) or inhibitors (Etomoxir). Oxygen consumption (OCR) was determined determined and expressed as values normalized to BSA-only treated cells (defined as 100%). * = $p<0.05$ from BSA-only treated cells; ** = $p < 0.001$ from palmitate WO/E, and no metformin treatment.

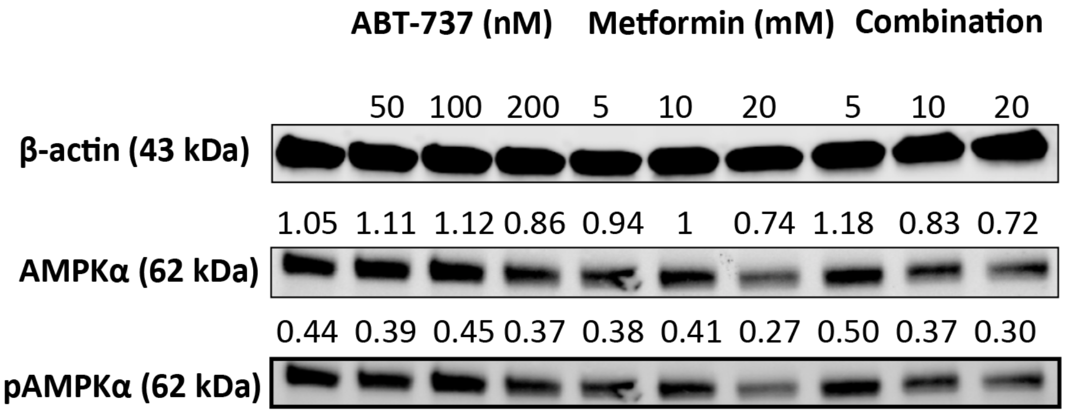


**Supplementary Figure S7: Metformin does not activate AMPK in OCI-AML3 leukemia cells.** OCI-AML3 cells were treated with treated with ABT-737, metformin or both in RPMI-1640 medium containing 10% fetal bovine serum for 2 hours. After incubation, the cells were counted and lysed to perform Western blot detection of AMPKa/β and phospho-AMPKa/β as well as β-actin as described in the Materials and Methods.

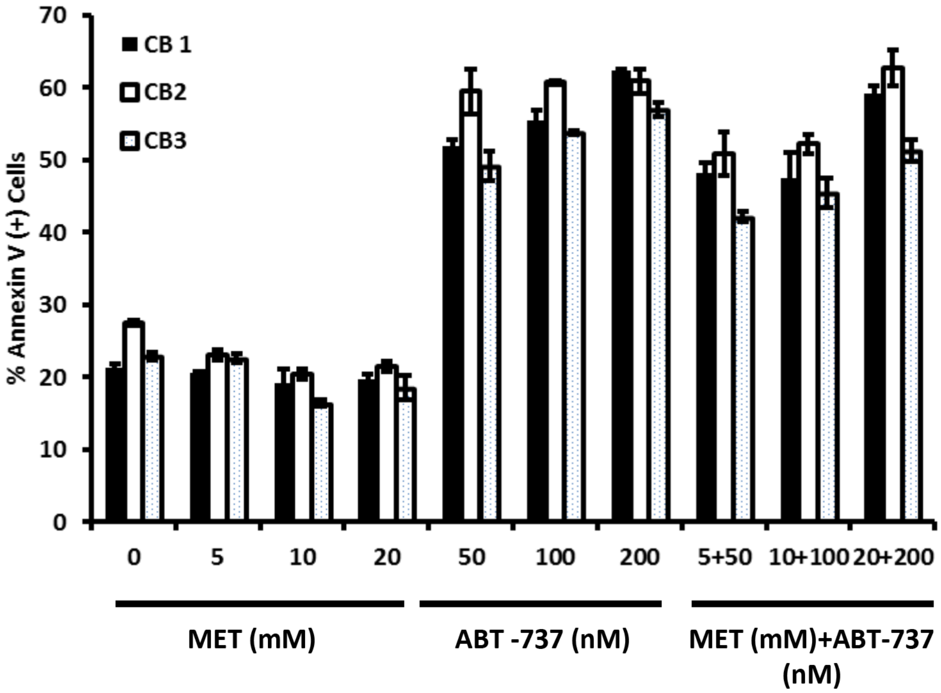


**Supplementary Figure S8: Metformin does not potentiate the cytotoxicity of ABT-737 in normal CD34-positive hematopoietic stem cells.** Cord blood mononuclear cells were exposed to ABT-737, metformin or both in RPMI-1640 medium containing 10% fetal bovine serum for 16 hours and viability and apoptosis were assessed by flow cytometry in the CD34-positive compartment as described in the Supplementary Methods.
